# The control of inflammation via the phosphorylation and dephosphorylation of tristetraprolin: a tale of two phosphatases

**DOI:** 10.1042/BST20160166

**Published:** 2016-10-19

**Authors:** Andrew R. Clark, Jonathan L.E. Dean

**Affiliations:** 1 Institute of Inflammation and Ageing, College of Medical and Dental Sciences, University of Birmingham, Birmingham B15 2TT, U.K.; 2 Kennedy Institute of Rheumatology, University of Oxford, Oxford OX3 7FY, U.K.

**Keywords:** dual-specificity phosphatases, MAPK p38, mRNA stability, PP2A, tristetraprolin

## Abstract

Twenty years ago, the first description of a tristetraprolin (TTP) knockout mouse highlighted the fundamental role of TTP in the restraint of inflammation. Since then, work from several groups has generated a detailed picture of the expression and function of TTP. It is a sequence-specific RNA-binding protein that orchestrates the deadenylation and degradation of several mRNAs encoding inflammatory mediators. It is very extensively post-translationally modified, with more than 30 phosphorylations that are supported by at least two independent lines of evidence. The phosphorylation of two particular residues, serines 52 and 178 of mouse TTP (serines 60 and 186 of the human orthologue), has profound effects on the expression, function and localisation of TTP. Here, we discuss the control of TTP biology via its phosphorylation and dephosphorylation, with a particular focus on recent advances and on questions that remain unanswered.

## Introduction

**Figure 1.**
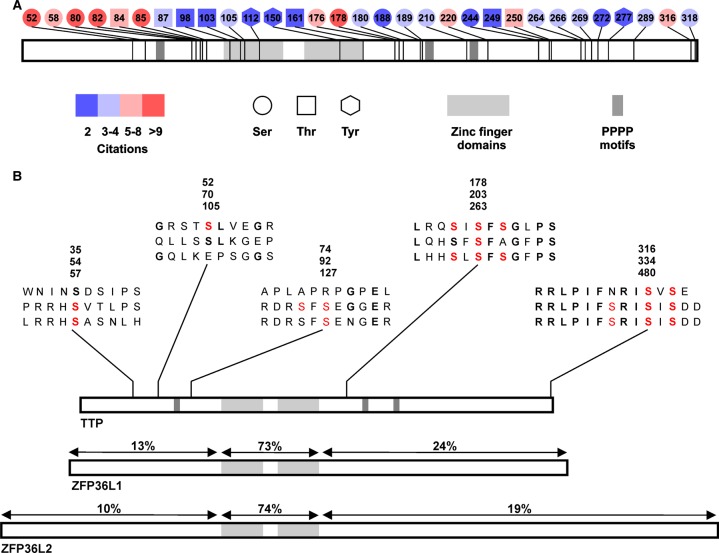
Sites of phosphorylation of TTP, and their conservation in other members of the ZFP36 family. (**A**) Schematic of documented phosphorylations of TTP, based on data from PhosphositePlus [39]. Phosphorylations supported by only one published source are omitted. There may be some bias in the coverage of TTP protein, due to the presence of putative phosphorylation sites in very large or small tryptic fragments, which may be poorly detected. The influence of specific phosphorylations on protein stability may also introduce bias, as discussed in the text. (**B**) Conservation and divergence of phosphorylation sites in members of the ZFP36 family. TTP, ZFP36L1 and ZFP36L2 proteins are illustrated schematically. Numbers above the N-terminal, zinc finger and C-terminal domains of ZFP36L1 and ZFP36L2 indicate % similarity with TTP itself. Peptide sequences of specific regions are indicated, in each case in the order (from top to bottom) TTP, ZFP36L1 and ZFP36L2. Co-ordinates of specific residues are indicated in the same order. Residues in bold are conserved between TTP and other members of the family. Residues in red are known to be phosphorylated *in vivo*.

Tristetraprolin (TTP) belongs to a small family of RNA-binding proteins, which has three members in most mammalian species but four in the mouse and rat. Its name derives from three dispersed stretches of four consecutive proline residues (Figure 1). Reflecting its independent discovery by several laboratories, it has also been named as 12-O-tetradecanoyl phorbol 13 acetate-inducible sequence 11a, G0/G1 switch gene 24, nuclear protein 475 and ZFP36 (zinc finger protein of 36 kDa). The protein is now almost universally known as TTP, whereas the correct systematic name for the corresponding gene is *Zfp36* in the mouse, *ZFP36* in man. The biology of TTP is very well reviewed elsewhere [1]. This review focuses on the role of phosphorylation and dephosphorylation of TTP in the regulation of inflammatory responses, unresolved controversies and questions that remain to be answered.

The first *Zfp36* gene knockout revealed a fundamental role of TTP in the constraint of inflammation [1–3]. Mice lacking TTP developed a spontaneous and pervasive inflammatory syndrome, including cachexia, dermatitis and erosive joint inflammation resembling rheumatoid arthritis (RA). Most symptoms were ascribed to increased stability of tumour necrosis factor (*Tnf*) mRNA and expression of TNF protein. Consequently, much research on TTP has focussed on myeloid cells, which are the principal sources of TNF *in vivo*. However, myeloid-specific disruption of the *Zfp36* gene did not reproduce the same pervasive syndrome. Mice lacking TTP in myeloid cells were healthy under normal conditions, but developed excessive inflammatory responses to challenge with lipopolysaccharide (LPS) [4,5]. These findings clearly establish that TTP functions in non-myeloid cells to inhibit inflammation. Although activation-induced TTP expression can be detected in several different cell types, relatively little is known about its function outside the myeloid lineage, other than in fibroblasts [6–8]. TNF sustains inflammation in part via its actions on fibroblasts [9], an effect that is modulated by fibroblast TTP [8,9a].

TTP is a sequence-specific mRNA-binding protein with a preferred binding site consisting of the heptameric sequence UAUUUAU [1]. The heptamer is a particular example of an element known as an adenylate/uridylate-rich element or ARE. Binding of TTP to RNA substrates is mediated by a central zinc finger domain (ZFD), in which two tandem zinc fingers each co-ordinate a single zinc ion via three cysteine and one histidine residues. A crystal structure has been solved for the RNA heptamer in complex with the ZFD of the TTP family member ZFP36L2 (ZFP36-like protein 2, otherwise known as butyrate response factor 2 or BRF-2) [10]. As the ZFD is highly conserved between the members of the family, it is highly likely that TTP recognises its targets in the same manner. The UAUUUAU heptamer and closely related sequences are frequently found in the 3′-untranslated regions (3′-UTRs) of mRNAs encoding cytokines, chemokines and other mediators of inflammation, growth factors, regulators of apoptosis and the cell cycle. A growing number of such mRNAs have been shown to be recognised and regulated by TTP [1]. However, *in silico* prediction of TTP targets remains difficult. Transcriptome-wide identification of targets has been performed using two related methods including PAR-CLIP (photoactivatable ribonucleoside-enhanced cross-linking and immunoprecipitation) and i-CLIP (individual nucleotide resolution cross-linking and immunoprecipitation) [11–13]. A user-friendly website integrates PAR-CLIP and i-CLIP data sets, providing rapid visualisation of transcriptome-wide TTP-binding sites, coupled with the analysis of differential mRNA expression in *Zfp36*−/− macrophages (http://ttp-atlas.univie.ac.at/index.php). Particularly where high confidence hits are selected, these two methods support the consensus heptamer as a preferred binding site for TTP *in vivo*. The absence of secondary structure appears to be an important determinant of binding [12], consistent with the structure of the ZFD in complex with an RNA substrate [10]. The presence of more than one consensus site also favours binding, perhaps reflecting co-operative interactions between TTP molecules [1,14]. TTP interactions were enriched near to the poly-(A) tail [11,13], possibly related to interactions between TTP and poly-(A)-binding proteins [15,16].

Like other members of its family, TTP is able to recruit several proteins or protein complexes that participate in mRNA turnover [15,17,18]. These include deadenylases, which catalyse the shortening of the 3′-poly-(A) tail; decapping enzymes, which remove the 7-methylguanylate cap from the 5′-end of mRNA and exonucleases that catalyse the degradation of the mRNA body from either the 5′-end or the 3′-end. In most cases, an obligate and rate-limiting step in the destruction of an mRNA is the removal of the poly-(A) tail [19]. Therefore, the interactions between TTP and deadenylases are likely to be highly relevant. In a simplified outline, binding of TTP to cognate sites in the 3′-UTRs of target mRNAs is followed by recruitment of deadenylases and shortening of the poly-(A) tail. When the poly-(A) tail becomes too short to support high-affinity binding of poly-(A)-binding proteins, destruction of the mRNA body is initiated by decapping of the 5′-end and rapid exonucleolytic degradation of the mRNA body. Hence, TTP target mRNAs tend to be abnormally stable in *Zfp36*−/− cells that lack TTP and highly unstable in *Zfp36aa/aa* cells expressing a constitutively active form of TTP known as TTP-AA, in which serines 52 and 178 (Ser-52 and Ser-178) are substituted by alanine residues [20] (see below). Some investigators have found TTP to regulate the expression of target mRNAs principally at the level of translation rather than mRNA stability [21–23]. More recently, TTP target mRNA abundance and translation were systematically studied using iCLIP, RNASeq and ribosome footprinting (RiboSeq) in *Zfp36*−/− macrophages reconstituted with GFP-TTP or GFP-TTP-AA [13]. This analysis suggested that TTP phosphorylation has greatest effects at the level of target mRNA stability. Relatively few transcripts were differentially expressed at the level of translation without evident changes of mRNA abundance. The reasons for the discrepancies between these observations are far from clear. It should be noted that similar controversy has surrounded the mitogen-activated protein kinase (MAPK) p38 pathway, and whether its post-transcriptional effects are chiefly at the level of mRNA stability or translation [24]. It may be relevant that the poly-(A) tail not only protects mRNA against degradation, but also promotes efficient mRNA translation via interactions between poly-(A)-binding proteins and 5′-cap-binding proteins [25]. One hypothesis is that target mRNA deadenylation may be uncoupled from degradation of the mRNA body under some conditions, or in a transcript-specific manner, such that the principal effects of MAPK p38 and TTP are to modulate translation rather than degradation. However, experimental evidence in support of this hypothesis is so far lacking.

## TTP as a phosphoprotein

In denaturing polyacrylamide gel electrophoresis, TTP appears either as a broad smear or as a ladder of discrete bands with apparent molecular mass between 40 and 55 kDa (the actual molecular mass of murine TTP being 33.6 kDa). Most or all of this variation in electrophoretic mobility is a consequence of phosphorylation, since phosphatase treatment of cell lysates collapsed the multiple forms of TTP to one or two bands of high mobility [26–29]. Conversely, in lysates from cells treated with phosphatase inhibitors, such as okadaic acid or calyculin A, TTP migrates with an apparent molecular mass of up to 62 kDa [27,30,31]. Only a fraction of this change in electrophoretic mobility can be attributed to the mere addition of mass. Even the phosphorylation of 30 residues of TTP would increase its mass by <3 kDa. However, it is evident that TTP can be very extensively phosphorylated *in vivo*.

Information about sites of phosphorylation of TTP has emerged both from high-throughput phosphoproteomic studies of tumour cells, macrophages and other cells [28,32–34] and from focussed experiments in which epitope-tagged human or mouse TTP was stably expressed in HEK293 human kidney cells [35–37], mouse 3T3 fibroblasts, hamster kidney [38] or a mouse macrophage cell line [20]. This information is very helpfully summarised on the PhosphositePlus web site (www.phosphosite.org) [39] and illustrated graphically in Figure 1. If all of the documented phosphorylations are accepted at face value, TTP is in the top 0.1% of phosphorylated proteins in the proteome in terms of the number of phosphorylations per unit mass. Making allowance for species divergence and technical differences, such as proteolytic cleavage strategies, targeted and untargeted approaches, generate a quite consistent picture of TTP phosphorylation in different cellular contexts. For consistency and clarity, we use murine TTP co-ordinates, even where the relevant experiments were performed using human TTP. The corresponding human co-ordinates can be read from PhosphositePlus. Phosphorylation of Ser-52 and Ser-178 is extremely well documented, although the former was not detected in focussed studies of transfected HEK293 cells [35,37], perhaps for technical reasons. As discussed below, TTP protein is stabilised via phosphorylation of Ser-52 and Ser-178. Therefore, TTP lacking these two phosphorylations may be low in abundance and consequently under-represented in phosphoproteomic analyses. A serine-, threonine- and proline-rich region N-terminal to the first zinc finger can be phosphorylated at Ser-80, Ser-82, Ser-85 and Thr-87. Additional clusters of phosphorylation sites are found in the region of the second and third tetraprolin motifs, and within the C-terminal domain of the protein. Mass spectrometric data indicate high stoichiometry of phosphorylation within these regions [20,36] and our unpublished data. Both targeted and untargeted approaches provide evidence for phosphorylation of Ser-316, very close to the C-terminus of TTP [20,28,32,37]. Finally, there is some evidence for phosphorylation of Tyr-112, Tyr-150 and Tyr-277. The majority of the sites discussed above are highly conserved between mammalian TTP orthologues, and several are also present in other members of the TTP family [35] (Figure 1B, discussed below).

## TTP function is modulated by phosphorylation

MAPK p38 plays a central role in the expression of many mediators of inflammatory responses [40]. To a large extent, it operates via the downstream kinase MAPK-activated protein kinase 2 (MK2), regulating target genes at a post-transcriptional level, inhibiting deadenylation, increasing mRNA stability and/or increasing its translational efficiency [41]. Tight regulation of MAPK p38 signalling is critical to prevent ectopic, excessive or unprovoked inflammation. An important negative feedback mechanism involves dual-specificity phosphatase 1 (DUSP1, also known as MAPK phosphatase 1 or MKP-1) [42–44]. DUSP1 is normally expressed at very low levels in resting cells. Pro-inflammatory stimuli increase its expression in an MAPK p38-dependent manner. It then dephosphorylates and inactivates MAPK p38 (as well as other MAPKs), helping to bring about the termination of the inflammatory response [42,43]. *Dusp1*−/− mice are healthy under normal conditions, but their dysregulated MAPK p38 responses to pro-inflammatory challenges are often fatal.

There is a considerable overlap between transcripts that are targeted by TTP and those that are post-transcriptionally regulated by MAPK p38. The pivotal role of TTP in MAPK p38-mediated post-transcriptional regulation of inflammatory responses was demonstrated by the failure of MAPK p38 inhibitors to decrease gene expression, or to destabilise target mRNAs, in *Zfp36*−/− cells [45,46]. MK2 efficiently phosphorylated TTP *in vitro* [26], and the major sites were subsequently identified as Ser-52, Ser-178 and Ser-316 [38]. A landmark paper then demonstrated that the MK2-mediated phosphorylation of Ser-52 and Ser-178 impaired the mRNA-destabilising activity of TTP [47]. This mRNA-stabilising effect was accompanied by an interaction of phosphorylated TTP with 14-3-3 proteins. These are a family of abundant, dimeric adaptor proteins that specifically recognise certain client phosphoproteins, helping to bring about changes in their structure, stability, function or subcellular localisation [48,49]. Phosphorylation of TTP and recruitment of 14-3-3 proteins could be enhanced by the treatment of cells with the somewhat unspecific phosphatase inhibitor, okadaic acid [27]. The identity of the cellular phosphatase or phosphatases responsible for the dephosphorylation of Ser-52 and Ser-178 is rather important. The best evidence to date implicates protein phosphatase 2A (PP2A), since siRNA-mediated knockdown of a catalytic subunit of PP2A increased TTP phosphorylation, 14-3-3 protein recruitment, expression of TNF and of a TNF 3′-UTR reporter mRNA [27]. The recruitment of 14-3-3 proteins is thought to antagonise PP2A-mediated dephosphorylation of TTP [27], with consequences that are discussed below.

Conflicting, although not mutually exclusive, mechanisms have been suggested to mediate the control of mRNA stability via MK2-mediated phosphorylation of TTP. According to one school of thought, TTP competes for RNA binding with HuR (human antigen R), a member of the embryonic lethal abnormal vision family of RNA-binding proteins [50–53]. HuR is generally considered as an mRNA-stabilising factor [54] and binds to RNA with specificity overlapping rather than identical with that of TTP. Phosphorylation of TTP is proposed to decrease its affinity for RNA and favour its displacement by HuR [23,55]. However, there is disagreement about the extent to which binding sites for TTP and HuR overlap [11,12]. High-resolution mapping by PAR-CLIP suggested that instances of direct competition between the two RNA-binding proteins may be rare [12]. Comparison of transcriptome-wide-binding sites of GFP-TTP and GFP-TTP-AA suggested that phosphorylation may decrease the affinity and/or specificity with which TTP binds to RNA [13]. However, the non-canonical TTP-binding sites described in that study were generally not found in another study using native rather than ectopically expressed TTP [12]. Tethered function assays provided evidence that MK2 modulates TTP function by means other than the regulation of RNA binding. When TTP was fused to bacteriophage coat protein MS2, it was able to direct the degradation of a reporter mRNA bearing MS2-binding sites. Activation of MK2 blocked the degradation of the reporter mRNA in a manner dependent on intact Ser-52 and Ser-178 sites [31]. In electrophoretic mobility shift assays, antibodies against 14-3-3 proteins strongly supershifted protein complexes with RNA probes containing the *Tnf* ARE [27 and our unpublished observations]. Since 14-3-3 proteins bind only weakly to unphosphorylated TTP, this observation implies that phosphorylated TTP can bind to an ARE with high affinity. More reductionist *in vitro* approaches using highly purified recombinant TTP suggest that MK2-mediated phosphorylation of TTP has no impact on its affinity for mRNA [56]. However, *in vivo* interactions with HuR, 14-3-3 and other unknown proteins may determine whether or not TTP binding occurs.

Several independent groups have reported that mammalian TTP promotes deadenylation of target mRNA by recruiting the carbon catabolite repressor protein 4-negative on TATA-less (CCR4–NOT) deadenylase complex, a large (1 mDa) complex containing at least 10 subunits [31,56–59]. The *Drosophila melanogaster* orthologue of TTP employs a similar mechanism to regulate the expression of anti-microbial products [60,61]. As the MAPK p38 pathway regulates mRNA stability at the level of poly-(A) tail length [62,63], an obvious hypothesis is that MK2-mediated phosphorylation of TTP impairs CCR4–NOT recruitment. TTP-dependent *in vitro* deadenylation and degradation of an ARE-containing reporter RNA were prevented by MK2-mediated phosphorylation of Ser-52 and Ser-178, which also inhibited the recruitment of the CCR4–NOT complex [56]. A 14-3-3 sequestering agent prevented the binding of 14-3-3 proteins to phosphorylated TTP, but did not destabilise the reporter RNA. This suggests that MK2-mediated phosphorylation of TTP may impair deadenylase recruitment directly rather than via binding of 14-3-3 proteins. However, another group also described inhibition of CCR4–NOT recruitment via the phosphorylation of Ser-52 and Ser-178, and implicated 14-3-3 binding in this process [31]. Two other groups described recruitment of CCR4–NOT1 via the C-terminal domain of TTP [57,58]. MK2-mediated phosphorylation of Ser-316 was shown to impair the interaction between TTP and CNOT1, a scaffold protein of the CCR4–NOT1 complex, thereby inhibiting deadenylation and degradation of TTP target transcripts [57,58]. These observations are not necessarily conflicting. As the CCR4–NOT1 complex is extremely large, there is potential for multiple contacts with TTP, and redundant, 14-3-3-dependent or independent mechanisms for regulation of the interaction by phosphorylation. Elucidation of the mechanisms and modulation of TTPs interaction with CCR4–NOT1 is not a trivial undertaking.

## TTP subcellular localisation is modulated by phosphorylation

Like many RNA-binding proteins, TTP shuttles between the nucleus and the cytoplasm. Nuclear export is mediated by CRM1 and dependent on a Leu-rich N-terminal region [64,65]. Both 14-3-3-dependent and -independent mechanisms maintain TTP in the cytoplasm [66]. The description of TTP as an almost exclusively cytoplasmic protein requires some qualification. At least in macrophages, TTP is likely to be phosphorylated at Ser-52 and Ser-178 as soon as it is generated. Although low amounts of endogenous, nuclear TTP can be detected [12], nuclear localisation has been most clearly demonstrated under somewhat artificial circumstances: ectopic expression in the absence of MAPK p38 activity [30,67,68], acute inhibition of MAPK p38 at the peak of TTP expression [30] or alanine substitution of Ser-52 and Ser-178 of endogenous TTP (ARC, in preparation). Together, these observations suggest that MAPK p38-dependent phosphorylation of Ser-52 and Ser-178 contributes to, but is not indispensible for, the localisation of TTP in the cytoplasm, at least in macrophages.

It remains unclear what biological function, if any, is served by TTP in the nucleus. A role in the regulation of nuclear polyadenylation of ARE-containing mRNAs was described in one report [69]. Large numbers of TTP-binding sites were identified by i-CLIP or PAR-CLIP in intronic RNA, and conventional RNA-IP confirmed binding of TTP to an excised intron, presumably in the nucleus [11,12]. The consequences of such interactions are not known. TTP has also been proposed to function as a transcriptional corepressor of nuclear hormone receptors or nuclear factor kappa-light-chain-enhancer of activated B cells (NF-κB) [70–72]. Alternatively, TTP may impair NF-κB function by preventing nuclear translocation of the p65 subunit [73–75], a phenomenon that would not necessarily require TTP to be present in the nucleus. Where non-canonical actions of TTP are proposed, which do not involve its binding to RNA, it is important to rule out indirect effects. For example, TTP may regulate the expression of components of the NF-κB signalling pathway or the expression of p65 itself [13].

Interruption of translation leads to the formation (or accretion) of granular cytoplasmic structures known as stress granules (SGs) and processing bodies (P bodies). Detailed description of these structures is beyond the scope of the present study, but reviewed extensively elsewhere [76–78]. SGs contain components of the translation machinery and are thought to be formed via aggregation of translationally stalled ribonucleoprotein complexes. P bodies are crucial sites of mRNA turnover that contain many components of the cellular mRNA degradation machinery, including the CCR4-NOT complex. The two discrete structures are dynamically linked and sometimes in contact with one another. TTP has been implicated in the traffic of ARE-containing mRNAs between P bodies and SGs, a process that may either determine or reflect decisions between mRNA storage, destruction and re-initiation of translation [78,79]. MK2-mediated phosphorylation of Ser-52 and Ser-178 was demonstrated to result in exclusion of TTP from SGs, accompanied by recruitment of 14-3-3 proteins and stabilisation of target mRNAs [47]. One might predict that phosphorylation of Ser-52 and Ser-178 also prevents localisation of TTP to P bodies, but to our knowledge this has not yet been demonstrated.

## TTP protein stability is modulated by phosphorylation

TTP protein is also stabilised in response to phosphorylation of Ser-52 and Ser-178 [21,30,80]. The description of TTP as a relatively stable protein [81] is therefore accurate under most circumstances, where TTP expression is accompanied by (indeed, dependent on) MAPK p38 activation. Lacking two sites of MK2-mediated phosphorylation, TTP-AA is constitutively degraded by the proteasome and therefore expressed at low levels [20,21,30]. If primary macrophages or RAW264.7 cells are stimulated with LPS for 2 h prior to the addition of an MAPK p38 inhibitor, pre-existing TTP is rapidly degraded in a manner that requires the activity of both phosphatase(s) and the proteasome [30].

In the majority of cases, targeting of proteins for destruction by the proteasome depends on the covalent addition of polyubiquitin chains to lysine residues. Unexpectedly, ubiquitination of TTP could not be detected, nor was TTP protected from proteasomal degradation by mutation of all five lysine residues [82]. These observations suggest an atypical mode of degradation of TTP protein. The only 3D structure of TTP protein solved to date is for the highly conserved central zinc finger region [83], and secondary structure prediction programmes all fail to identify any stable structure in the N-terminal and C-terminal domains [82] (JLED, unpublished data). The unstructured nature of the majority of TTP protein is consistent with proteasomal degradation via a default degradation pathway shared with other largely unstructured proteins [84]. A key question is how the phosphorylation of Ser-52 and Ser-178 acts to stabilise TTP protein. Since a common feature of 14-3-3 proteins is that they impose secondary structure upon client phosphoproteins [48,49], an obvious hypothesis is that 14-3-3-mediated imposition of stable structure allows TTP protein to escape degradation-by-default. Gueydan and colleagues report that the addition of recombinant 14-3-3 proteins failed to prevent degradation of purified TTP *in vitro* [82]. It would arguably be more relevant (though less easy) to ask whether 14-3-3 proteins are necessary for the stabilisation of phosphorylated TTP.

## A working model and an experimental validation

**Figure 2.**
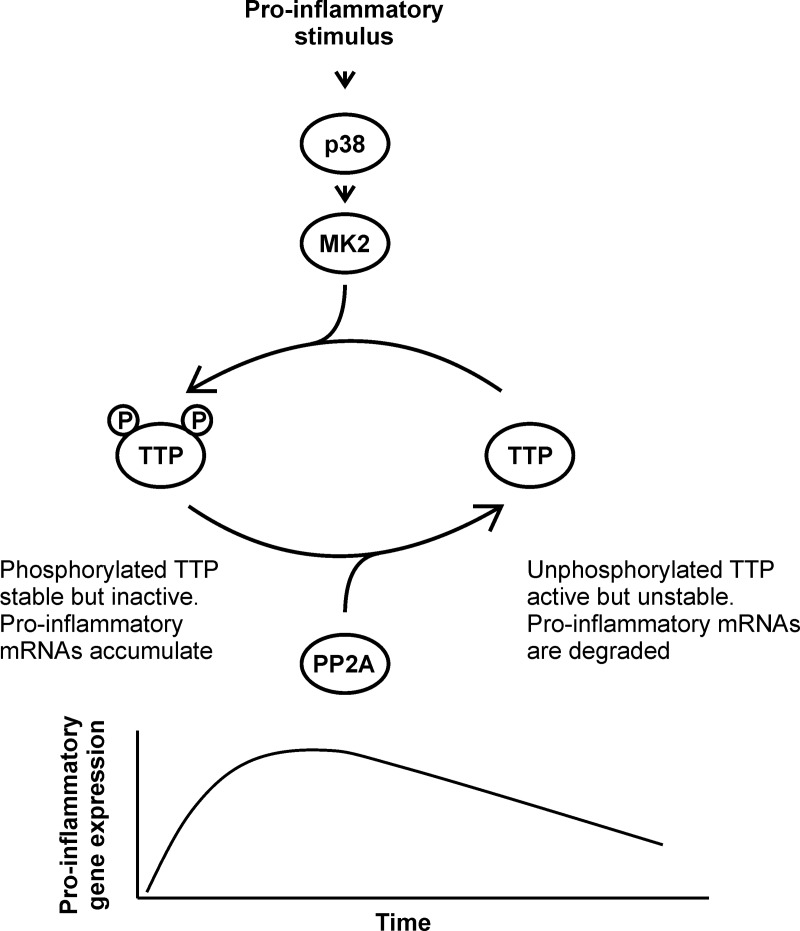
Schematic model of the regulation of TTP expression and function by the MAPK p38 signalling pathway. The dynamic equilibrium between phosphorylation and dephosphorylation of TTP controls the switch between the on- and off-phases of pro-inflammatory gene expression. Only Ser-52 and Ser-178 phosphorylations are indicated.

The regulation of both TTP expression and function by the MAPK p38 pathway effectively couples the activation and resolution phases of an inflammatory response. A working model of this process is presented in Figure 2. During the early phase of the response to a pro-inflammatory agonist, such as LPS, strong MAPK p38 activity promotes the expression of TTP at the levels of transcription, mRNA stability, mRNA translation and protein stability. Although direct experimental evidence is lacking, the TTP that accumulates under these conditions is thought to be phosphorylated at Ser-52, Ser-178 and Ser-316, therefore inactive as an mRNA destabiliser or translation suppressor. Efficient expression of inflammatory mediators is therefore possible. As MAPK p38 activity declines, the balance between phosphorylation and dephosphorylation of these sites shifts in favour of the latter. The accumulated pool of inert TTP then becomes active and can promote the off-phase of the response, blocking further translation, promoting mRNA decay or both. This signal-dependent post-transcriptional regulation of ARE-containing mRNAs contributes to the complex programme of temporally tuned gene expression, in which the influence of TTP increases with time [4,12,14,20].

Illustrating the physiological significance of MAPK p38-mediated regulation of TTP function, we recently used homologous recombination to generate a knockin mouse strain, in which Ser-52 and Ser-178 codons of the endogenous *Zfp36* locus were substituted by alanine codons [20]. We refer to the mutated locus as *Zfp36aa* and the altered protein product as TTP-AA. Homozygous *Zfp36aa/aa* mice proved healthy and fertile with no evident phenotype under standard maintenance conditions. As mentioned above, TTP-AA was expressed weakly (being constitutively degraded by the proteasome), but it constitutively destabilised target mRNAs and strongly inhibited the expression of several inflammatory mediators. When challenged by intraperitoneal injection of LPS, *Zfp36aa/aa* mice were protected against the subsequent cytokine storm and organ damage, reflecting dramatically reduced serum levels of many inflammatory cytokines. For example, in these experiments, the expression of interleukin 6 (IL-6) was ∼200-fold lower in *Zfp36aa/aa* than in *Zfp36*+/+ mice (ARC, in preparation). In a highly robust experimental model of inflammatory arthritis, *Zfp36aa/aa* mice were completely protected, developing absolutely no symptoms of disease [9a]. They also demonstrated decreased pathogenic responses in some experimental models of pulmonary inflammation (Phil Hansbro and Alaina Ammit, personal communication). Other experimental models of inflammatory pathology remain to be tested.

We hypothesised that DUSP1 regulates inflammatory responses by modulating the phosphorylation state, and hence the activity, of TTP. By combining *Dusp1*−/− and *Zfp36aa/aa* genotypes, it was demonstrated that harmful, dysregulated inflammatory responses in the absence of DUSP1 were largely dependent on intact Ser-52 and Ser-178 residues of TTP [85]. Certain genes, for example, *Tnf*, *Cxcl1* and *Cxcl2*, were regulated by DUSP1 exclusively via TTP phosphorylation. Their expression was strongly elevated in *Dusp1*−/− macrophages and equally strongly diminished in *Zfp36aa/aa* macrophages. In double genetically modified macrophages, expression of these genes remained low, indicating that dysregulated MAPK p38 signalling could enhance gene expression only if TTP could be phosphorylated and inactivated. DUSP1 controlled many other genes in part via TTP and in part via other mechanisms that have not been identified, but probably include effects on transcription. The regulation of TTP function by the two phosphatases, DUSP1 and PP2A, is illustrated schematically in Figure 2. DUSP1 indirectly regulates the inactivation of TTP, whereas PP2A directly mediates the activation of TTP.

Several groups have reported that TTP is not only regulated by DUSP1, but also regulates the expression of DUSP1 [13,86–91]. Interaction of TTP with the *Dusp1* 3′-UTR was also confirmed in iCLIP and PAR-CLIP studies [12,13]. This suggests the existence of a homeostatic feedback mechanism by which TTP may regulate its own function. Elevated TTP activity would be predicted to decrease DUSP1 expression and enhance the activity of MAPK p38, ultimately promoting TTP inactivation. Consistent with this concept, the kinetics of MAPK p38 activation were altered in retrovirally transduced macrophages expressing GFP-TTP-AA [13]. In contrast, no changes in MAPK p38 activation profile were observed in macrophages expressing TTP-AA from the endogenous *Zfp36* promoter [20]. Nevertheless, this putative homeostatic mechanism may be physiologically relevant and merits further investigation.

There is a great deal more that remains puzzling or unknown about the phosphorylation and dephosphorylation of TTP. The remainder of this review focuses on a few unanswered questions.

## Are phosphorylation and dephosphorylation of TTP linked to pathogenesis?

TTP protein was strongly expressed at sites of inflammation in cardiovascular disease and a mouse model thereof, where it was suggested to play an anti-inflammatory role at both transcriptional and post-transcriptional levels [75]. TTP was also abundant in the inflamed RA synovium, prompting the authors to ask why it failed to down-regulate the expression of TNF and other inflammatory mediators [92]. Such observations might be re-interpreted in light of the coupled stabilisation and inactivation of TTP due to phosphorylation of Ser-52 and Ser-178. We hypothesise that TTP can accumulate in a phosphorylated and inactive form at sites of prolonged inflammation, and that its inactivity contributes to the establishment of chronicity. In support of this hypothesis, we found TTP to be co-localised with active MK2 in the cytoplasm of RA synovial macrophages [9a]. Constitutive MAPK p38 activation in tumour-associated macrophages was accompanied by accumulation of inactive TTP, contributing to overexpression of several inflammatory mediators [22]. However, the phosphorylation state of Ser-52 and Ser-178 at inflammatory or oncogenic lesions has not yet been directly demonstrated. Both we [20] and others [47] have generated phospho-specific antibodies against TTP, but so far these have not proved amenable to approaches such as immunofluorescence. More sophisticated methods may be required to determine whether TTP is phosphorylated and inactivated at sites of chronic inflammation.

TTP and other members of its family are putative tumour suppressors, whose expression is diminished or absent from various cancers [53,93–98]. Inhibition of tumourigenesis may occur at several different levels; for example, down-regulation of cell cycle regulators and proto-oncogenes, growth factors, inflammatory cytokines and proteases that support tumour growth, vascularisation or metastasis. Phosphorylation and inactivation rather than loss of TTP has also been implicated in tumour development [99]. Potent anti-proliferative effects were ascribed to a mutant form of TTP, in which eight phospho-acceptor sites, including Ser-52 and Ser-178, were substituted by non-phosphorylatable alanines [100]. It would be interesting to test responses of the *Zfp36aa/aa* mouse in experimental models of tumourigenesis, particularly those in which TTP targets, such as cyclooxygenase 2, have been implicated as pathogenic factors.

## Why are Ser-52 and Ser-178 evolutionarily conserved?

**Figure 3.**
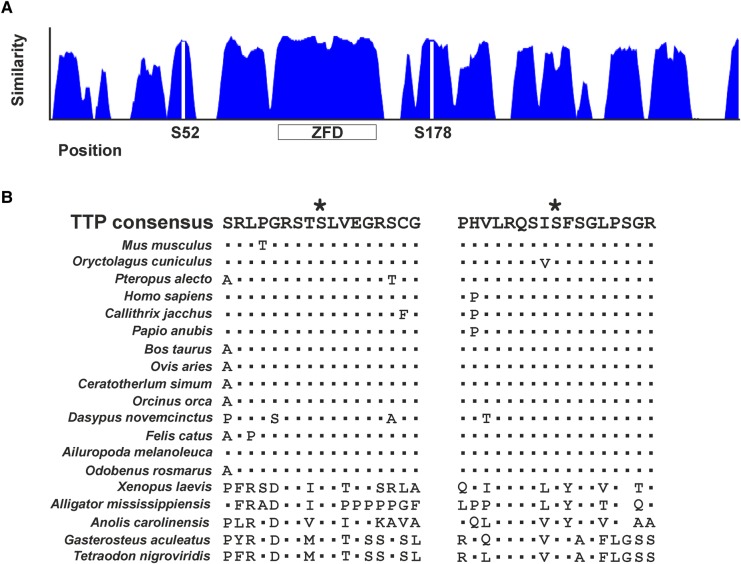
Conservation of phosphorylation sites Ser-52 and Ser-178. (**A**) Plot of similarity of TTP protein sequences of 47 vertebrate species, of which 2 are amphibian, 2 reptilian, 3 bony fish and the remainder mammals. The positions of residues corresponding to Ser-52 and Ser-178 are shown by vertical white bars. ZFD, zinc finger domain. (**B**) Sequences surrounding putative MK2 phosphorylation sites in selected TTP orthologues. The consensus is derived from the 47 vertebrate species mentioned above. Identical residues are indicated by dots. *Xenopus laevis* is an amphibian; *Alligator mississippiensis* and *Anolis carolinensis* are reptiles; *Gasterosteus aculeatus* and *Tetraodon nigroviridis* are fish. All other species are mammals.

As described above, *Zfp36aa/aa* mice were protected against excessive inflammatory responses in experimental models of endotoxemia, RA and pulmonary inflammation. Moreover, their ability to mount protective immune responses against a model pathogen was not significantly impaired [20]. Yet in the real world outside the laboratory, the phosphorylation of TTP appears to be under strong selective pressure. The sequences surrounding serines 52 and 178 are highly conserved amongst mammals (Figure 3). Although there is predictably greater sequence divergence in fish, amphibian and reptile TTP, the phospho-acceptor sites themselves are conserved. They are predicted to be recognised and phosphorylated by MK2, although this remains to be experimentally tested. The implication is that the control of TTP function via phosphorylation arose early during vertebrate evolution and has been maintained by natural selection. In turn, this implies that *Zfp36aa/aa* mice have some selective disadvantages that have not yet come to light under laboratory conditions. We speculate that this relates to the innate immune response to pathogens more virulent than those tested so far, organisms that generate the strongest possible selective pressure. Interestingly, a virulent strain of the intracellular pathogen *Francisella tularensis* promotes increased apoptosis of infected macrophages, accompanied by increased stability of *Il1b* mRNA and secretion of IL-1β protein. This response was linked to sustained phosphorylation of serine 178 of TTP [34]. *Bacillus anthracis* has a more evasive strategy, using lethal toxin (Le-Tx) to cleave MAPK kinases, silence MAPK signalling cascades and reduce innate immune responses. Le-Tx was shown to favour the formation of P bodies and promote destabilisation of IL-8 mRNA in a TTP-dependent manner [101]. These observations suggest that TTP and its phosphorylation sites are at the battlefront of an evolutionary war between hosts and pathogens, finely balanced between excessive and insufficient innate immune responses. It would be interesting to determine whether *Zfp36aa/aa* macrophages are more or less susceptible than wild-type macrophages to infection by these two pathogens.

## What are the mediators and the consequences of other phosphorylations of TTP?

Surprisingly, little is known about sites of phosphorylation of TTP other than Ser-52 and Ser-178. Several of the sites are followed by proline residues and are candidates for phosphorylation by MAPKs or other proline-directed kinases. Indeed, recombinant TTP was found to be efficiently phosphorylated by extracellular signal-regulated kinase (ERK), cJun N-terminal kinase (JNK) or MAPK p38 *in vitro* [102,103]. We found that ERK phosphorylated several sites of recombinant TTP *in vitro*, and that an inhibitor of the ERK pathway decreased the phosphorylation of these sites in LPS-treated RAW264.7 cells without diminishing TTP protein levels (ARC, unpublished). These observations may be significant because ERK is thought to exert post-transcriptional effects via TTP [30,104,105]. Glycogen synthase kinase 3b, protein kinases A and Cμ also phosphorylated recombinant TTP *in vitro*, but sites were not identified [103]. Mediators of tyrosine phosphorylations have not been identified.

The electrophoretic mobility of TTP was strongly influenced by phosphorylation of serines 189, 210 and 220 [37]. As discussed above, such changes in mobility cannot be attributed to simple acquisition of mass. It is thought that large, phosphorylation-dependent changes in electrophoretic mobility generally involve *cis*–*trans*-isomerisation at proline residues adjacent to phospho-acceptor sites, which are catalysed by Pin1 (protein interacting with never in mitosis 1) or other prolyl isomerases [106]. Although there is no obvious link between electrophoretic mobility and cellular function, prolyl isomerisation can impose significant reorganisation of protein structure, affecting interaction with partner proteins, nucleic acids or other substrates. There is therefore potential for these mobility-associated phosphorylations to impact TTP function. Pin1 modulates the function of other RNA-binding proteins [107], but physical and functional interactions with TTP have not yet been described. Finally, it is tempting to speculate that the addition of multiple phosphates adjacent to or within the ZFD of TTP alters its affinity for RNA substrates.

## How and where is the dephosphorylation of TTP performed?

Even if PP2A is correctly identified as the phosphatase solely responsible for dephosphorylation of Ser-52 and Ser-178, the matter does not rest there. PP2A functions as a heterotrimer between a structural subunit, a regulatory subunit and a catalytic subunit [108,109]. Catalytic and structural subunits can each be encoded by two different genes. Regulatory subunits are encoded by at least 24 different genes, many of which give rise to different proteins due to alternative splicing or translation initiation. It is the B subunit that dictates the specificity of the trimeric holoenzyme for phosphoprotein substrates. The activity of PP2A holoenzyme is negatively regulated by a large family of inhibitors, some of which appear to have specificity for PP2A isoforms and are themselves regulated by phosphorylation. Even without taking into account post-translational modifications of PP2A subunits, this system generates huge diversity, allowing PP2A to participate in the regulation of a broad range of cellular functions. At this point, we do not know how PP2A is targeted to TTP, and which subunit(s) of PP2A are involved in the interaction. We do not know where the dephosphorylation of TTP occurs, and whether PP2A isoforms are present in SGs, P bodies or polysomes.

## Therapeutic implications of the regulation of TTP by PP2A and DUSP1

The MAPK p38 signalling pathway plays a fundamental role in the regulation of inflammatory responses and was long considered as a promising target for novel anti-inflammatory drugs [40]. In fact, the first MAPK p38 inhibitors were discovered in a screen for compounds that inhibited macrophage expression of TNF [110]. However, clinical trials of several different classes of MAPK p38 inhibitors yielded disappointing results, including anti-inflammatory effects that were not sustained [111]. The unanticipated negative results have almost put a halt to this line of translational enterprise, although the underlying reasons are not clear [112]. In macrophages, prolonged inhibition of MAPK p38 prevented the expression of TTP protein [26] and failed to destabilise TTP target mRNAs [46,113]. Therefore, the complex role of MAPK p38 in controlling both the expression and the activity of TTP may help to explain why chronic inhibition of this pathway does not exert the expected anti-inflammatory effects.

An intriguing question is whether PP2A might be therapeutically targeted to promote the dephosphorylation and activation of TTP. Several compounds have been shown to promote PP2A function, usually by disrupting interactions between the phosphatase and its inhibitory protein partners [108,109]. One such compound, a sphingolipid known as AAL(s), exerted TTP-dependent anti-inflammatory effects in an airway epithelial cell line [114,115]. AAL(s) and another PP2A agonist, the apolipoprotein E-derived peptide COG1410, exerted protective effects and prevented bone erosion in an experimental model of RA [9a]. *In vitro*, COG1410 decreased the expression of TNF in *Zfp36*+/+ macrophages, but not in *Zfp36aa/aa* macrophages. It also increased the electophoretic mobility and decreased the expression of wild-type TTP protein, but had neither of these effects on TTP-AA [9a]. These observations are consistent with COG1410 exerting anti-inflammatory effects by promoting the dephosphorylation and increasing the activity of TTP. They constitute no more than proof of principle for the concept of therapeutic targeting of PP2A in inflammation. Because of its multiple cellular functions, indiscriminate activation of PP2A is a risky strategy. For this reason, it is important to understand the physical and functional interaction between PP2A and TTP in greater detail. An added complication is that elevated PP2A activity and a consequent increase in TTP function has been implicated in age-related impairment of immunoglobulin class switching in B cells [116,117]. It is therefore possible that stimulation of PP2A could have undesired effects on adaptive immunity.

Reflecting its central role in the regulation of innate immune responses, DUSP1 is targeted by many endogenous immunoregulators as a means of promoting or suppressing inflammatory responses [42,118]. For example, glucocorticoids (GCs) exert anti-inflammatory effects in part by enhancing and prolonging the expression of DUSP1, and thereby curtailing MAPK p38 activity [119–124]. GCs [125] and a variety of other anti-inflammatory agonists have been reported to increase the expression of TTP. An emerging concept is that anti-inflammatory agonists may exert their effects not only by increasing TTP expression, but also by targeting DUSP1 to promote the activation of TTP [126]. Both *Dusp1*−/− and *Zfp36aa/aa* mouse strains will be useful for further exploration of this concept.

## Lessons from relatives of TTP

Two broadly expressed TTP family members are ZFP36L1 (otherwise known as butyrate response factor 1 or BRF-1; TPA-inducible sequence 11B or TIS11B; B-cell early response gene of 36 kDa or Berg36) and ZFP36L2 (also known as BRF-2 or TIS11D). A fourth family member, ZFP36L3, appears to be expressed only in rodent placenta and is not discussed here [127]. Similarity between TTP, ZFP36L1 and ZFP36L2 is highest (>70%) within the zinc finger RNA-binding domains and drops to 10–24% outside of these domains (Figure 1B). Two additional short stretches of high similarity are discussed below. The members of the ZFP36 family have very similar RNA-binding specificities, and they recruit the same complexes of enzymes to regulate the degradation and/or translation of their targets [17,18,128]. However, disruption of the murine *Zfp36*, *Zfp36l1* or *Zfp36l2* genes has very different consequences. The pro-inflammatory effects of *Zfp36* gene disruption have been discussed above. *Zfp36l1* gene disruption is embryonic lethal due to defects in placental function [129,130]. *Zfp36l2* gene disruption causes perinatal mortality associated with defective haematopoiesis [131]. Conditional knockouts have demonstrated critical roles of ZFP36L1 and ZFP36L2 in the development of both B- and T-cell lineages [132,133]. It is unclear how much overlap exists between functions and mRNA targets of ZFP36 family members. To some extent, differences in the phenotypes of knockouts may be explained by different tissue-specific and developmental patterns of expression or different kinetics of expression in response to cell stimulation [91]. However, there are circumstances in which two or more family members are present. For example, both TTP and ZFP36L1 are expressed and phosphorylated in LPS-treated macrophages [28]. The pro- and anti-inflammatory macrophage phenotypes arising from the absence of TTP or the expression of a constitutively active form are striking. In contrast, there is little evidence that ZFP36L1 plays an important role in restraining macrophage inflammatory responses [134]. The nature and extent of redundancy between these proteins is puzzling.

Another intriguing question is whether members of the ZFP36 family differ in their regulation by phosphorylation. Although there are no published phosphoproteomic studies focussing on ZFP36L1 or ZFP36L2, some information on their phosphorylation can be obtained from high-throughput studies. As a rule, phosphorylations of TTP seem not to be shared by its relatives. In some cases, the sites themselves are not conserved. For example, the extensively phosphorylated, proline-rich domain between residues 80 and 90 of TTP is absent from ZFP36L1 and ZFP36L2. In other cases, potential phosphorylation sites are conserved but adjacent residues may not favour their phosphorylation. An example is serine 220, which is followed by a proline residue in TTP. In ZFP36L1 or ZFP36L2, the equivalent serine residue is followed by alanine or serine, which will preclude phosphorylation by proline-directed kinases. The sequence surrounding Ser-52 is not well conserved in ZFP36L1 and ZFP36L2 (Figure 1B). On the other hand, certain prominent phosphorylations appear to be specific to ZFP36L1 and ZFP36L2. These are at Ser-54 and Ser-92 of ZFP36L1 and at Ser-57 and Ser-127 of ZFP36L2. The phospho-acceptor sites and/or surrounding residues are not conserved in TTP (Figure 1B). There are two protein regions in which both amino acid sequence and phosphorylation are conserved between TTP and its relatives. The first is centred around Ser-178 (Figure 1B). There is good evidence of phosphorylation of the corresponding residues Ser-203 in ZFP36L1 and Ser-263 in ZFP36L2. The second conserved phosphorylation domain is at the C-terminus (Figure 1B). ZFP36L1 can be phosphorylated at Ser-334 and Ser-336, ZFP36L2 at Ser-480 and Ser-482, which correspond to Ser-316 and Ser-318 of TTP.

The phosphorylation of ZFP36L1 at Ser-92 and Ser-203 promotes recruitment of 14-3-3 proteins and regulates both ZFP36L1 protein stability and mRNA-destabilising activity [135–137]. This is remarkably similar to the regulation of TTP stability and function via the phosphorylation of Ser-52 and Ser-178. However, while Ser-203 of ZFP36L1 corresponds to Ser-178 of TTP, Ser-92 does not correspond to Ser-52 (Figure 1B). We speculate that evolutionary acquisition of novel phospho-acceptor sites has allowed the proteins to diverge in terms of the exact location of the sites used, while retaining the same basic mechanism of regulation by phosphorylation. While one study implicated MK2 in the phosphorylation of ZFP36L1 at Ser-92 and Ser-203 [136], others implicated the kinase Akt, downstream of phosphatidylinositol 3-kinase (PI3K) [135,137,138]. The fact that these two distinct kinases may phosphorylate the same residues in ZFP36L1 is unsurprising, given that their substrate specificities overlap. It is likely that MK2 and/or Akt regulate the function of ZFP36L2, although to our knowledge this has not been demonstrated. Another intriguing question is whether the expression and function of TTP may also be regulated by PI3K-Akt.

MK2-mediated phosphorylation of Ser-316 and Ser-318 in the C-terminus of TTP is thought to stabilise TTP targets by impairing the recruitment of CCR4–NOT [58]. The C-terminal domain has been strongly conserved throughout the duplication and evolution of the ZFP36 family, and is clearly recognisable even in oyster and lamprey orthologues [139]. Regulation of function via phosphorylation of the C-terminus may therefore have been a property of the ancestral ZFP36 protein. In the cases of ZFP36L1 and ZFP36L2, phosphorylation can be mediated by p90 ribosomal S6 kinase, which is downstream of ERK [140]. The emerging picture of the relationship between kinases and ZFP36 family members appears increasingly complex, as individual phospho-acceptor sites may be targeted by more than one kinase pathway, and more than one family member may be subjected to phosphorylation in a given cell type.

## Conclusion

TTP plays a vital role in orchestrating the finely tuned, temporally precise responses of macrophages to pro-inflammatory stimuli. Work from several laboratories has contributed to a detailed understanding of certain elements of TTP function. It has been demonstrated, using cell transfections, *in vitro* assays and genetically modified mouse strains, that the MAPK p38 pathway mediates the phosphorylation of key residues of TTP to modulate both its expression and its function. This constitutes an elegant system for linking the initiation of an inflammatory response to its resolution. Stronger or more prolonged activation of MAPK p38 will generate a more robust induction of inflammatory mediators, but it will also generate a larger pool of dormant TTP, which is then ready to promote the destruction of inflammatory mRNAs as soon as MAPK p38 activity declines. While we may admire the elegance of the biology, we need to remember quite how much remains unknown about TTP. For example, most of the above discussion has centred on just two sites of phosphorylation of TTP. We do not yet understand the influences of other signalling pathways via the same sites or the importance of the phosphorylation of ortholgous sites on other members of the TTP family. Most importantly, there are at least 28 additional sites of TTP phosphorylation about which we know next to nothing.

## Abbreviations

3′-UTRs, 3′-untranslated regions; ARE, adenylate-/uridylate-rich element; CCR4-NOT, carbon catabolite repressor protein 4-negative on TATA-less; DUSP1, dual-specificity phosphatase 1; ERK, extracellular signal-regulated kinase; GCs, glucocorticoids; HuR, human antigen R; i-CLIP, individual nucleotide resolution cross-linking and immunoprecipitation; Le-Tx, lethal toxin; LPS, lipopolysaccharide; MAPK, mitogen-activated protein kinase; MK2, MAPK-activated protein kinase 2; NF-κB, nuclear factor kappa-light-chain-enhancer of activated B cells; P bodies, processing bodies; PAR-CLIP, photoactivatable ribonucleoside-enhanced cross-linking and immunoprecipitation; PI3K, phosphatidylinositol 3-kinase; Pin1, protein interacting with never in mitosis 1; PP2A, protein phosphatase 2A; RA, rheumatoid arthritis; Ser-52 and Ser-178, serines 52 and 178; SGs, stress granules; TNF, tumour necrosis factor; TTP, tristetraprolin; ZFD, zinc finger domain; ZFP36L2, ZFP36-like protein 2.

## Funding

Andy Clark's research on TTP was funded by Arthritis Research UK [Programme Grant 19614] and the Medical Research Council [Project Grant G0800207].

## Competing Interests

The Authors declare that there are no competing interests associated with the manuscript.

## References

[1] Brooks S.A. and Blackshear P.J. (2013) Tristetraprolin (TTP): interactions with mRNA and proteins, and current thoughts on mechanisms of action. Biochim. Biophys. Acta, Gene Regul. Mech. 1829, 666–679 doi:10.1016/j.bbagrm.2013.02.003 PMC3752887 23428348

[2] Taylor G.A., Carballo E., Lee D.M., Lai W.S., Thompson M.J., Patel D.D. et al. (1996) A pathogenetic role for TNFα in the syndrome of cachexia, arthritis, and autoimmunity resulting from tristetraprolin (TTP) deficiency. Immunity 4, 445–454 doi:10.1016/S1074-7613(00)80411-2 8630730

[3] Carballo E., Gilkeson G.S. and Blackshear P.J. (1997) Bone marrow transplantation reproduces the tristetraprolin-deficiency syndrome in recombination activating gene-2 (–/–) mice. Evidence that monocyte/macrophage progenitors may be responsible for TNFalpha overproduction. J. Clin. Invest. 100, 986–995 doi:10.1172/JCI119649 9276715 PMC508273

[4] Kratochvill F., Machacek C., Vogl C., Ebner F., Sedlyarov V., Gruber A.R. et al. (2011) Tristetraprolin-driven regulatory circuit controls quality and timing of mRNA decay in inflammation. Mol. Syst. Biol. 7, 560 doi:10.1038/msb.2011.93 22186734 PMC3737733

[5] Qiu L.-Q., Stumpo D.J. and Blackshear P.J. (2012) Myeloid-specific tristetraprolin deficiency in mice results in extreme lipopolysaccharide sensitivity in an otherwise minimal phenotype. J. Immunol. 188, 5150–5159 doi:10.4049/jimmunol.1103700 22491258 PMC3345041

[6] Lai W.S., Parker J.S., Grissom S.F., Stumpo D.J. and Blackshear P.J. (2006) Novel mRNA targets for tristetraprolin (TTP) identified by global analysis of stabilized transcripts in TTP-deficient fibroblasts. Mol. Cell. Biol. 26, 9196–9208 doi:10.1128/MCB.00945-06 17030620 PMC1698545

[7] Chen X., Wei Z., Wang W., Yan R., Xu X. and Cai Q. (2012) Role of RNA-binding protein tristetraprolin in tumor necrosis factor-α mediated gene expression. Biochem. Biophys. Res. Commun. 428, 327–332 doi:10.1016/j.bbrc.2012.09.033 22995314

[8] Qiu L.-Q., Lai W.S., Bradbury A., Zeldin D.C. and Blackshear P.J. (2015) Tristetraprolin (TTP) coordinately regulates primary and secondary cellular responses to proinflammatory stimuli. J. Leukoc. Biol. 97, 723–736 doi:10.1189/jlb.3A0214-106R 25657290 PMC4370050

[9] Armaka M., Apostolaki M., Jacques P., Kontoyiannis D.L., Elewaut D. and Kollias G. (2008) Mesenchymal cell targeting by TNF as a common pathogenic principle in chronic inflammatory joint and intestinal diseases. J. Exp. Med. 205, 331–337 doi:10.1084/jem.20070906 18250193 PMC2271010

[9a] Ross E.A., Naylor A.J., O'Neil J.D., Crowley T., Ridley M.L., Crowe J. et al. (2016) Treatment of inflammatory arthritis via targeting of Tristetraprolin, a master regulator of pro-inflammatory gene expression. Ann. Rheum. Dis. in press. 10.1136/annrheumdis-2016-209424 PMC5446007 27597652

[10] Hudson B.P., Martinez-Yamout M.A., Dyson H.J. and Wright P.E. (2004) Recognition of the mRNA AU-rich element by the zinc finger domain of TIS11d. Nat. Struct. Mol. Biol. 11, 257–264 doi:10.1038/nsmb738 14981510

[11] Mukherjee N., Jacobs N.C., Hafner M., Kennington E.A., Nusbaum J.D., Tuschl T. et al. (2014) Global target mRNA specification and regulation by the RNA-binding protein ZFP36. Genome Biol. 15, R12 doi:10.1186/gb-2014-15-1-r12 24401661 PMC4053807

[12] Sedlyarov V., Fallmann J., Ebner F., Huemer J., Sneezum L., Ivin M. et al. (2016) Tristetraprolin binding site atlas in the macrophage transcriptome reveals a switch for inflammation resolution. Mol. Syst. Biol. 12, 868 doi:10.15252/msb.20156628 27178967 PMC4988506

[13] Tiedje C., Diaz-Muñoz M.D., Trulley P., Ahlfors H., Laaß K., Blackshear P.J. et al. (2016) The RNA-binding protein TTP is a global post-transcriptional regulator of feedback control in inflammation. Nucleic Acids Res. doi:10.1093/nar/gkw474 PMC5009735 27220464

[14] Rabani M., Raychowdhury R., Jovanovic M., Rooney M., Stumpo D.J., Pauli A. et al. (2014) High-resolution sequencing and modeling identifies distinct dynamic RNA regulatory strategies. Cell 159, 1698–1710 doi:10.1016/j.cell.2014.11.015 25497548 PMC4272607

[15] Tiedje C., Kotlyarov A. and Gaestel M. (2010) Molecular mechanisms of phosphorylation-regulated TTP (tristetraprolin) action and screening for further TTP-interacting proteins. Biochem. Soc. Trans. 38, 1632–1637 doi:10.1042/BST0381632 21118139

[16] Rowlett R.M., Chrestensen C.A., Schroeder M.J., Harp M.G., Pelo J.W., Shabanowitz J. et al. (2008) Inhibition of tristetraprolin deadenylation by poly(A) binding protein. Am. J. Physiol. Gastrointest. Liver Physiol. 295, G421–G430 doi:10.1152/ajpgi.00508.2007 18467502 PMC2536786

[17] Lykke-Andersen J. and Wagner E. (2005) Recruitment and activation of mRNA decay enzymes by two ARE-mediated decay activation domains in the proteins TTP and BRF-1. Genes Dev. 19, 351–361 doi:10.1101/gad.1282305 15687258 PMC546513

[18] Franks T.M. and Lykke-Andersen J. (2007) TTP and BRF proteins nucleate processing body formation to silence mRNAs with AU-rich elements. Genes Dev. 21, 719–735 doi:10.1101/gad.1494707 17369404 PMC1820945

[19] Garneau N.L., Wilusz J. and Wilusz C.J. (2007) The highways and byways of mRNA decay. Nat. Rev. Mol. Cell Biol. 8, 113–126 doi:10.1038/nrm2104 17245413

[20] Ross E.A., Smallie T., Ding Q., O'Neil J.D., Cunliffe H.E., Tang T. et al. (2015) Dominant suppression of inflammation via targeted mutation of the mRNA destabilizing protein tristetraprolin. J. Immunol. 195, 265–276 doi:10.4049/jimmunol.1402826 26002976 PMC4472942

[21] Hitti E., Iakovleva T., Brook M., Deppenmeier S., Gruber A.D., Radzioch D. et al. (2006) Mitogen-activated protein kinase-activated protein kinase 2 regulates tumor necrosis factor mRNA stability and translation mainly by altering tristetraprolin expression, stability, and binding to adenine/uridine-rich element. Mol. Cell. Biol. 26, 2399–2407 doi:10.1128/MCB.26.6.2399-2407.2006 16508014 PMC1430282

[22] Kratochvill F., Gratz N., Qualls J.E., Van De Velde L.-A., Chi H., Kovarik P. et al. (2015) Tristetraprolin limits inflammatory cytokine production in tumor-associated macrophages in an mRNA decay-independent manner. Cancer Res. 75, 3054–3064 doi:10.1158/0008-5472.CAN-15-0205 26183929 PMC4526390

[23] Tiedje C., Ronkina N., Tehrani M., Dhamija S., Laass K., Holtmann H. et al. (2012) The p38/MK2-driven exchange between tristetraprolin and HuR regulates AU-rich element-dependent translation. PLoS Genet. 8, e1002977 doi:10.1371/journal.pgen.1002977 23028373 PMC3459988

[24] Clark A.R., Dean J.L.E. and Saklatvala J. (2003) Post-transcriptional regulation of gene expression by mitogen-activated protein kinase p38. FEBS Lett. 546, 37–44 doi:10.1016/S0014-5793(03)00439-3 12829234

[25] Smith R.W.P., Blee T.K.P. and Gray N.K. (2014) Poly(A)-binding proteins are required for diverse biological processes in metazoans. Biochem. Soc. Trans. 42, 1229–1237 doi:10.1042/BST20140111 25110030 PMC4128646

[26] Mahtani K.R., Brook M., Dean J.L.E., Sully G., Saklatvala J. and Clark A.R. (2001) Mitogen-activated protein kinase p38 controls the expression and posttranslational modification of tristetraprolin, a regulator of tumor necrosis factor alpha mRNA stability. Mol. Cell. Biol. 21, 6461–6469 doi:10.1128/MCB.21.9.6461-6469.2001 11533235 PMC99793

[27] Sun L., Stoecklin G., Van Way S., Hinkovska-Galcheva V., Guo R.-F., Anderson P. et al. (2007) Tristetraprolin (TTP)-14-3-3 complex formation protects TTP from dephosphorylation by protein phosphatase 2a and stabilizes tumor necrosis factor-α mRNA. J. Biol. Chem. 282, 3766–3777 doi:10.1074/jbc.M607347200 17170118

[28] Weintz G., Olsen J.V., Frühauf K., Niedzielska M., Amit I., Jantsch J. et al. (2010) The phosphoproteome of toll-like receptor-activated macrophages. Mol. Syst. Biol. 6, 371 doi:10.1038/msb.2010.29 20531401 PMC2913394

[29] Cao H. (2004) Expression, purification, and biochemical characterization of the antiinflammatory tristetraprolin: a zinc-dependent mRNA binding protein affected by posttranslational modifications. Biochemistry 43, 13724–13738 doi:10.1021/bi049014y 15504035 PMC1351390

[30] Brook M., Tchen C.R., Santalucia T., McIlrath J., Arthur J.S.C., Saklatvala J. et al. (2006) Posttranslational regulation of tristetraprolin subcellular localization and protein stability by p38 mitogen-activated protein kinase and extracellular signal-regulated kinase pathways. Mol. Cell. Biol. 26, 2408–2418 doi:10.1128/MCB.26.6.2408-2418.2006 16508015 PMC1430283

[31] Clement S.L., Scheckel C., Stoecklin G. and Lykke-Andersen J. (2011) Phosphorylation of tristetraprolin by MK2 impairs AU-rich element mRNA decay by preventing deadenylase recruitment. Mol. Cell. Biol. 31, 256–266 doi:10.1128/MCB.00717-10 21078877 PMC3019984

[32] Zhou H., Di Palma S., Preisinger C., Peng M., Polat A.N., Heck A.J.R. et al. (2013) Toward a comprehensive characterization of a human cancer cell phosphoproteome. J. Proteome Res. 12, 260–271 doi:10.1021/pr300630k 23186163

[33] Huttlin E.L., Jedrychowski M.P., Elias J.E., Goswami T., Rad R., Beausoleil S.A. et al. (2010) A tissue-specific Atlas of mouse protein phosphorylation and expression. Cell 143, 1174–1189 doi:10.1016/j.cell.2010.12.001 21183079 PMC3035969

[34] Nakayasu E.S., Tempel R., Cambronne X.A., Petyuk V.A., Jones M.B., Gritsenko M.A. et al. (2013) Comparative phosphoproteomics reveals components of host cell invasion and post-transcriptional regulation during *Francisella* infection. Mol. Cell. Proteomics 12, 3297–3309 doi:10.1074/mcp.M113.029850 23970565 PMC3820940

[35] Cao H., Deterding L.J. and Blackshear P.J. (2007) Phosphorylation site analysis of the anti-inflammatory and mRNA-destabilizing protein tristetraprolin. Expert Rev. Proteomics 4, 711–726 doi:10.1586/14789450.4.6.711 18067411 PMC2674331

[36] Cao H., Deterding L.J. and Blackshear P.J. (2014) Identification of a major phosphopeptide in human tristetraprolin by phosphopeptide mapping and mass spectrometry. PLoS ONE 9, e100977 doi:10.1371/journal.pone.0100977 25010646 PMC4091943

[37] Cao H., Deterding L.J., Venable J.D., Kennington E.A., Yates J.R., III Tomer K.B. et al. (2006) Identification of the anti-inflammatory protein tristetraprolin as a hyperphosphorylated protein by mass spectrometry and site-directed mutagenesis. Biochem. J. 394, 285–297 doi:10.1042/BJ20051316 16262601 PMC1386027

[38] Chrestensen C.A., Schroeder M.J., Shabanowitz J., Hunt D.F., Pelo J.W., Worthington M.T. et al. (2004) MAPKAP kinase 2 phosphorylates tristetraprolin on in vivo sites including Ser178, a site required for 14-3-3 binding. J. Biol. Chem. 279, 10176–10184 doi:10.1074/jbc.M310486200 14688255

[39] Hornbeck P.V., Zhang B., Murray B., Kornhauser J.M., Latham V. and Skrzypek E. (2015) Phosphositeplus, 2014: mutations, PTMs and recalibrations. Nucleic Acids Res. 43, D512–D520 doi:10.1093/nar/gku1267 25514926 PMC4383998

[40] Arthur J.S. and Ley S.C. (2013) Mitogen-activated protein kinases in innate immunity. Nat. Rev. Immunol. 13, 679–692 doi:10.1038/nri3495 23954936

[41] Gaestel M. (2013) What goes up must come down: molecular basis of MAPKAP kinase 2/3-dependent regulation of the inflammatory response and its inhibition. Biol. Chem. 394, 1301–1315 doi:10.1515/hsz-2013-0197 23832958

[42] Abraham S.M. and Clark A.R. (2006) Dual-specificity phosphatase 1: a critical regulator of innate immune responses. Biochem. Soc. Trans. 34, 1018–1023 doi:10.1042/BST0341018 17073741

[43] Lang R., Hammer M. and Mages J. (2006) DUSP meet immunology: dual specificity MAPK phosphatases in control of the inflammatory response. J. Immunol. 177, 7497–7504 doi:10.4049/jimmunol.177.11.7497 17114416

[44] Bode J.G., Ehlting C. and Häussinger D. (2012) The macrophage response towards LPS and its control through the p38MAPK–STAT3 axis. Cell. Signal. 24, 1185–1194 doi:10.1016/j.cellsig.2012.01.018 22330073

[45] Carballo E., Cao H., Lai W.S., Kennington E.A., Campbell D. and Blackshear P.J. (2001) Decreased sensitivity of tristetraprolin-deficient cells to p38 inhibitors suggests the involvement of tristetraprolin in the p38 signaling pathway. J. Biol. Chem. 276, 42580–42587 doi:10.1074/jbc.M104953200 11546803 PMC1351389

[46] Tudor C., Marchese F.P., Hitti E., Aubareda A., Rawlinson L., Gaestel M. et al. (2009) The p38 MAPK pathway inhibits tristetraprolin-directed decay of interleukin-10 and pro-inflammatory mediator mRNAs in murine macrophages. FEBS Lett. 583, 1933–1938 doi:10.1016/j.febslet.2009.04.039 19416727 PMC4798241

[47] Stoecklin G., Stubbs T., Kedersha N., Wax S., Rigby W.F.C., Blackwell T.K. et al. (2004) MK2-induced tristetraprolin:14-3-3 complexes prevent stress granule association and ARE-mRNA decay. EMBO J. 23, 1313–1324 doi:10.1038/sj.emboj.7600163 15014438 PMC381421

[48] Yaffe M.B. (2002) How do 14-3-3 proteins work? – Gatekeeper phosphorylation and the molecular anvil hypothesis. FEBS Lett. 513, 53–57 doi:10.1016/S0014-5793(01)03288-4 11911880

[49] Bustos D.M. (2012) The role of protein disorder in the 14-3-3 interaction network. Mol. Biosyst. 8, 178–184 doi:10.1039/C1MB05216K 21947246

[50] Al-Souhibani N., Al-Ghamdi M., Al-Ahmadi W. and Khabar K.S.A. (2014) Posttranscriptional control of the chemokine receptor CXCR4 expression in cancer cells. Carcinogenesis 35, 1983–1992 doi:10.1093/carcin/bgu080 24692066 PMC4146410

[51] Griseri P. and Pagès G. (2014) Control of pro-angiogenic cytokine mRNA half-life in cancer: the role of AU-rich elements and associated proteins. J. Interferon Cytokine Res. 34, 242–254 doi:10.1089/jir.2013.0140 24697202

[52] Soler D.M., Ghosh A., Chen F. and Shneider B.L. (2014) A single element in the 3′-UTR of the apical sodium-dependent bile acid transporter controls both stabilization and destabilization of mRNA. Biochem. J. 462, 547–553 doi:10.1042/BJ20140070 24946903 PMC5338459

[53] Young L.E., Sanduja S., Bemis-Standoli K., Pena E.A., Price R.L. and Dixon D.A. (2009) The mRNA binding proteins HuR and tristetraprolin regulate cyclooxygenase 2 expression during colon carcinogenesis. Gastroenterology 136, 1669–1679 doi:10.1053/j.gastro.2009.01.010 19208339 PMC3742387

[54] Brennan C.M. and Steitz J.A. (2001) HuR and mRNA stability. Cell. Mol. Life Sci. 58, 266–277 doi:10.1007/PL00000854 11289308 PMC11146503

[55] Zhao W., Liu M., D'Silva N.J. and Kirkwood K.L. (2011) Tristetraprolin regulates interleukin-6 expression through p38 MAPK-dependent affinity changes with mRNA 3′-untranslated region. J. Interferon Cytokine Res. 31, 629–637 doi:10.1089/jir.2010.0154 21457063 PMC3151618

[56] Marchese F.P., Aubareda A., Tudor C., Saklatvala J., Clark A.R. and Dean J.L. (2010) MAPKAP kinase 2 blocks tristetraprolin-directed mRNA decay by inhibiting CAF1 deadenylase recruitment. J. Biol. Chem. 285, 27590–27600 doi:10.1074/jbc.M110.136473 20595389 PMC2934626

[57] Sandler H., Kreth J., Timmers H.T.M. and Stoecklin G. (2011) Not1 mediates recruitment of the deadenylase Caf1 to mRNAs targeted for degradation by tristetraprolin. Nucleic Acids Res. 39, 4373–4386 doi:10.1093/nar/gkr011 21278420 PMC3105394

[58] Fabian M.R., Frank F., Rouya C., Siddiqui N., Lai W.S., Karetnikov A. et al. (2013) Structural basis for the recruitment of the human CCR4-NOT deadenylase complex by tristetraprolin. Nat. Struct. Mol. Biol. 20, 735–739 doi:10.1038/nsmb.2572 23644599 PMC4811204

[59] Shi J.-X., Li J.-S., Hu R., Shi Y., Su X., Li Q. et al. (2014) CNOT7/hCAF1 is involved in ICAM-1 and IL-8 regulation by tristetraprolin. Cell. Signal. 26, 2390–2396 doi:10.1016/j.cellsig.2014.07.020 25038453

[60] Vindry C., Lauwers A., Hutin D., Soin R., Wauquier C., Kruys V. et al. (2012) dTIS11 protein-dependent polysomal deadenylation is the key step in AU-rich element-mediated mRNA decay in *Drosophila* cells. J. Biol. Chem. 287, 35527–35538 doi:10.1074/jbc.M112.356188 22932903 PMC3471692

[61] Choi Y.-J., Lai W.S., Fedic R., Stumpo D.J., Huang W., Li L. et al. (2014) The *Drosophila* Tis11 protein and its effects on mRNA expression in flies. J. Biol. Chem. 289, 35042–35060 doi:10.1074/jbc.M114.593491 25342740 PMC4271196

[62] Dean J.L.E., Sarsfield S.J., Tsounakou E. and Saklatvala J. (2003) p38 mitogen-activated protein kinase stabilizes mRNAs that contain cyclooxygenase-2 and tumor necrosis factor AU-rich elements by inhibiting deadenylation. J. Biol. Chem. 278, 39470–39476 doi:10.1074/jbc.M306345200 12882963

[63] Winzen R., Gowrishankar G., Bollig F., Redich N., Resch K. and Holtmann H. (2004) Distinct domains of AU-rich elements exert different functions in mRNA destabilization and stabilization by p38 mitogen-activated protein kinase or HuR. Mol. Cell. Biol. 24, 4835–4847 doi:10.1128/MCB.24.11.4835-4847.2004 15143177 PMC416423

[64] Murata T., Yoshino Y., Morita N. and Kaneda N. (2002) Identification of nuclear import and export signals within the structure of the zinc finger protein TIS11. Biochem. Biophys. Res. Commun. 293, 1242–1247 doi:10.1016/S0006-291X(02)00363-7 12054509

[65] Phillips R.S., Ramos S.B.V. and Blackshear P.J. (2002) Members of the tristetraprolin family of tandem CCCH zinc finger proteins exhibit CRM1-dependent nucleocytoplasmic shuttling. J. Biol. Chem. 277, 11606–11613 doi:10.1074/jbc.M111457200 11796723

[66] Johnson B.A., Stehn J.R., Yaffe M.B. and Blackwell T.K. (2002) Cytoplasmic localization of tristetraprolin involves 14-3-3-dependent and -independent mechanisms. J. Biol. Chem. 277, 18029–18036 doi:10.1074/jbc.M110465200 11886850

[67] DuBois R.N., McLane M.W., Ryder K., Lau L.F. and Nathans D. (1990) A growth factor-inducible nuclear protein with a novel cysteine/histidine repetitive sequence. J. Biol. Chem. 265, 19185–19191 PMID: 1699942

[68] Taylor G.A., Thompson M.J., Lai W.S. and Blackshear P.J. (1996) Mitogens stimulate the rapid nuclear to cytosolic translocation of tristetraprolin, a potential zinc-finger transcription factor. Mol. Endocrinol. 10, 140–146 doi:10.1210/mend.10.2.8825554 8825554

[69] Su Y.-L., Wang S.-C., Chiang P.-Y., Lin N.-Y., Shen Y.-F., Chang G.-D. et al. (2012) Tristetraprolin inhibits poly(A)-tail synthesis in nuclear mRNA that contains AU-rich elements by interacting with poly(A)-binding protein nuclear 1. PLoS ONE 7, e41313 doi:10.1371/journal.pone.0041313 22844456 PMC3406032

[70] Barrios-García T., Gómez-Romero V., Tecalco-Cruz Á., Valadéz-Graham V. and León-Del-Río A. (2016) Nuclear tristetraprolin acts as a corepressor of multiple steroid nuclear receptors in breast cancer cells. Mol. Genet. Metab. Rep. 7, 20–26 doi:10.1016/j.ymgmr.2016.02.004 27114912 PMC4832087

[71] Liang J., Lei T., Song Y., Yanes N., Qi Y. and Fu M. (2009) RNA-destabilizing factor tristetraprolin negatively regulates NF-κB signaling. J. Biol. Chem. 284, 29383–29390 doi:10.1074/jbc.M109.024745 19738286 PMC2785570

[72] Xu L., Ning H., Gu L., Wang Q., Lu W., Peng H. et al. (2015) Tristetraprolin induces cell cycle arrest in breast tumor cells through targeting AP-1/c-Jun and NF-κB pathway. Oncotarget 6, 41679–41691 doi:10.18632/oncotarget.6149 26497679 PMC4747181

[73] Gu L., Ning H., Qian X., Huang Q., Hou R., Almourani R. et al. (2013) Suppression of IL-12 production by tristetraprolin through blocking NF-kcyB nuclear translocation. J. Immunol. 191, 3922–3930 doi:10.4049/jimmunol.1300126 23997224 PMC3788611

[74] Schichl Y.M., Resch U., Hofer-Warbinek R. and de Martin R. (2009) Tristetraprolin impairs NF-κB/p65 nuclear translocation. J. Biol. Chem. 284, 29571–29581 doi:10.1074/jbc.M109.031237 19654331 PMC2785590

[75] Zhang H., Taylor W.R., Joseph G., Caracciolo V., Gonzales D.M., Sidell N. et al. (2013) mRNA-binding protein ZFP36 is expressed in atherosclerotic lesions and reduces inflammation in aortic endothelial cells. Arterioscler. Thromb. Vasc. Biol. 33, 1212–1220 doi:10.1161/ATVBAHA.113.301496 23559629 PMC3844532

[76] Anderson P., Kedersha N. and Ivanov P. (2015) Stress granules, P-bodies and cancer. Biochim. Biophys. Acta, Gene Regul. Mech. 1849, 861–870 doi:10.1016/j.bbagrm.2014.11.009 PMC4457708 25482014

[77] Decker C.J. and Parker R. (2012) P-bodies and stress granules: possible roles in the control of translation and mRNA degradation. Cold Spring Harb. Perspect. Biol. 4, a012286 doi:10.1101/cshperspect.a012286 22763747 PMC3428773

[78] Kedersha N., Stoecklin G., Ayodele M., Yacono P., Lykke-Andersen J., Fitzler M.J. et al. (2005) Stress granules and processing bodies are dynamically linked sites of mRNP remodeling. J. Cell Biol. 169, 871–884 doi:10.1083/jcb.200502088 15967811 PMC2171635

[79] Murata T., Morita N., Hikita K., Kiuchi K. and Kaneda N. (2005) Recruitment of mRNA-destabilizing protein TIS11 to stress granules is mediated by its zinc finger domain. Exp. Cell Res. 303, 287–299 doi:10.1016/j.yexcr.2004.09.031 15652343

[80] Werno C., Schmid T., Schnitzer S.E., Peters K., Milke L. and Brüne B. (2010) A combination of hypoxia and lipopolysaccharide activates tristetraprolin to destabilize proinflammatory mRNAs such as tumor necrosis factor-α. Am. J. Pathol. 177, 1104–1112 doi:10.2353/ajpath.2010.091212 20639458 PMC2928945

[81] Cao H., Tuttle J.S. and Blackshear P.J. (2004) Immunological characterization of tristetraprolin as a low abundance, inducible, stable cytosolic protein. J. Biol. Chem. 279, 21489–21499 doi:10.1074/jbc.M400900200 15010466 PMC1351392

[82] Ngoc L.V., Wauquier C., Soin R., Bousbata S., Twyffels L., Kruys V. et al. (2014) Rapid proteasomal degradation of posttranscriptional regulators of the TIS11/tristetraprolin family is induced by an intrinsically unstructured region independently of ubiquitination. Mol. Cell. Biol. 34, 4315–4328 doi:10.1128/MCB.00643-14 25246635 PMC4248748

[83] Amann B.T., Worthington M.T. and Berg J.M. (2003) A Cys^3^His zinc-binding domain from Nup475/tristetraprolin: a novel fold with a disklike structure. Biochemistry 42, 217–221 doi:10.1021/bi026988m 12515557

[84] Asher G., Reuven N. and Shaul Y. (2006) 20S proteasomes and protein degradation ‘by default’. Bioessays 28, 844–849 doi:10.1002/bies.20447 16927316

[85] Smallie T., Ross E.A., Ammit A.J., Cunliffe H.E., Tang T., Rosner D.R. et al. (2015) Dual-specificity phosphatase 1 and tristetraprolin cooperate to regulate macrophage responses to lipopolysaccharide. J. Immunol. 195, 277–288 doi:10.4049/jimmunol.1402830 26019272 PMC4472943

[86] Bros M., Wiechmann N., Besche V., Art J., Pautz A., Grabbe S. et al. (2010) The RNA binding protein tristetraprolin influences the activation state of murine dendritic cells. Mol. Immunol. 47, 1161–1170 doi:10.1016/j.molimm.2009.11.002 19945750

[87] Emmons J., Townley-Tilson W.H.D., Deleault K.M., Skinner S.J., Gross R.H., Whitfield M.L. et al. (2008) Identification of TTP mRNA targets in human dendritic cells reveals TTP as a critical regulator of dendritic cell maturation. RNA 14, 888–902 doi:10.1261/rna.748408 18367721 PMC2327351

[88] Lin N.-Y., Lin C.-T. and Chang C.-J. (2008) Modulation of immediate early gene expression by tristetraprolin in the differentiation of 3T3-L1 cells. Biochem. Biophys. Res. Commun. 365, 69–74 doi:10.1016/j.bbrc.2007.10.119 17971298

[89] Lin N.-Y., Lin T.-Y., Yang W.-H., Wang S.-C., Wang K.-T., Su Y.-L. et al. (2012) Differential expression and functional analysis of the tristetraprolin family during early differentiation of 3T3-L1 preadipocytes. Int. J. Biol. Sci. 8, 761–777 doi:10.7150/ijbs.4036 22701344 PMC3371571

[90] Shah S., Mostafa M.M., McWhae A., Traves S.L. and Newton R. (2016) Negative feed-forward control of tumor necrosis factor (TNF) by tristetraprolin (ZFP36) is limited by the mitogen-activated protein kinase phosphatase, dual-specificity phosphatase 1 (DUSP1): implications for regulation by glucocorticoids. J. Biol. Chem. 291, 110–125 doi:10.1074/jbc.M115.697599 26546680 PMC4697149

[91] Wang K.-T., Wang H.-H., Wu Y.-Y., Su Y.-L., Chiang P.-Y., Lin N.-Y. et al. (2015) Functional regulation of Zfp36l1 and Zfp36l2 in response to lipopolysaccharide in mouse RAW264.7 macrophages. J. Inflamm. 12, 689 doi:10.1186/s12950-015-0088-x PMC4502546 26180518

[92] Brooks S.A., Connolly J.E., Diegel R.J., Fava R.A. and Rigby W.F.C. (2002) Analysis of the function, expression, and subcellular distribution of human tristetraprolin. Arthritis Rheum. 46, 1362–1370 doi:10.1002/art.10235 12115244

[93] Brennan S.E., Kuwano Y., Alkharouf N., Blackshear P.J., Gorospe M. and Wilson G.M. (2009) The mRNA-destabilizing protein tristetraprolin is suppressed in many cancers, altering tumorigenic phenotypes and patient prognosis. Cancer Res. 69, 5168–5176 doi:10.1158/0008-5472.CAN-08-4238 19491267 PMC2724875

[94] Ross C.R., Brennan-Laun S.E. and Wilson G.M. (2012) Tristetraprolin: roles in cancer and senescence. Ageing Res. Rev. 11, 473–484 doi:10.1016/j.arr.2012.02.005 22387927 PMC3376680

[95] Rounbehler R.J., Fallahi M., Yang C., Steeves M.A., Li W., Doherty J.R. et al. (2012) Tristetraprolin impairs Myc-induced lymphoma and abolishes the malignant state. Cell 150, 563–574 doi:10.1016/j.cell.2012.06.033 22863009 PMC3422762

[96] Sanduja S., Blanco F.F., Young L.E., Kaza V. and Dixon D.A. (2012) The role of tristetraprolin in cancer and inflammation. Front. Biosci. 17, 174–188 doi:10.2741/3920 PMC3461960 22201737

[97] Van Tubergen E.A., Banerjee R., Liu M., Vander Broek R.V., Light E., Kuo S. et al. (2013) Inactivation or loss of TTP promotes invasion in head and neck cancer via transcript stabilization and secretion of MMP9, MMP2, and IL-6. Clin. Cancer Res. 19, 1169–1179 doi:10.1158/1078-0432.CCR-12-2927 23349315 PMC3594656

[98] Hitti E., Bakheet T., Al-Souhibani N., Moghrabi W., Al-Yahya S., Al-Ghamdi M. et al. (2016) Systematic analysis of AU-rich element expression in cancer reveals common functional clusters regulated by key RNA-binding proteins. Cancer Res. 76, 4068–4080 doi:10.1158/0008-5472.CAN-15-3110 27197193

[99] Suswam E., Li Y., Zhang X., Gillespie G.Y., Li X., Shacka J.J. et al. (2008) Tristetraprolin down-regulates interleukin-8 and vascular endothelial growth factor in malignant glioma cells. Cancer Res. 68, 674–682 doi:10.1158/0008-5472.CAN-07-2751 18245466

[100] Suswam E.A., Shacka J.J., Walker K., Lu L., Li X., Si Y. et al. (2013) Mutant tristetraprolin: a potent inhibitor of malignant glioma cell growth. J. Neurooncol. 113, 195–205 doi:10.1007/s11060-013-1112-8 23525947 PMC3679321

[101] Chow E.M.C., Batty S. and Mogridge J. (2010) Anthrax lethal toxin promotes dephosphorylation of TTP and formation of processing bodies. Cell. Microbiol. 12, 557–568 doi:10.1111/j.1462-5822.2009.01418.x 19995385 PMC2859114

[102] Cao H., Dzineku F. and Blackshear P.J. (2003) Expression and purification of recombinant tristetraprolin that can bind to tumor necrosis factor-α mRNA and serve as a substrate for mitogen-activated protein kinases. Arch. Biochem. Biophys. 412, 106–120 doi:10.1016/S0003-9861(03)00012-2 12646273 PMC1351391

[103] Cao H. and Lin R. (2008) Phosphorylation of recombinant tristetraprolin in vitro. Protein J. 27, 163–169 doi:10.1007/s10930-007-9119-7 18071886 PMC2674330

[104] Bourcier C., Griseri P., Grepin R., Bertolotto C., Mazure N. and Pages G. (2011) Constitutive ERK activity induces downregulation of tristetraprolin, a major protein controlling interleukin8/CXCL8 mRNA stability in melanoma cells. Am. J. Physiol. Cell Physiol. 301, C609–C618 doi:10.1152/ajpcell.00506.2010 21593445

[105] Essafi-Benkhadir K., Pouysségur J. and Pagès G. (2010) Implication of the ERK pathway on the post-transcriptional regulation of VEGF mRNA stability. Methods Mol. Biol. 661, 451–469 doi:10.1007/978-1-60761-795-2_28 20812001

[106] Liou Y.-C., Zhou X.Z. and Lu K.P. (2011) Prolyl isomerase Pin1 as a molecular switch to determine the fate of phosphoproteins. Trends Biochem. Sci. 36, 501–514 doi:10.1016/j.tibs.2011.07.001 21852138 PMC3185210

[107] Shen Z.-J. and Malter J.S. (2015) Regulation of AU-rich element RNA binding proteins by phosphorylation and the prolyl isomerase Pin1. Biomolecules 5, 412–434 doi:10.3390/biom5020412 25874604 PMC4496679

[108] Lambrecht C., Haesen D., Sents W., Ivanova E. and Janssens V. (2013) Structure, regulation, and pharmacological modulation of PP2A phosphatases. Methods Mol. Biol. 1053, 283–305 doi:10.1007/978-1-62703-562-0_17 23860660

[109] Sangodkar J., Farrington C.C., McClinch K., Galsky M.D., Kastrinsky D.B. and Narla G. (2016) All roads lead to PP2A: exploiting the therapeutic potential of this phosphatase. FEBS J. 283, 1004–1024 doi:10.1111/febs.13573 26507691 PMC4803620

[110] Lee J.C., Laydon J.T., McDonnell P.C., Gallagher T.F., Kumar S., Green D. et al. (1994) A protein kinase involved in the regulation of inflammatory cytokine biosynthesis. Nature 372, 739–746 doi:10.1038/372739a0 7997261

[111] Genovese M.C. (2009) Inhibition of p38: has the fat lady sung? Arthritis Rheum. 60, 317–320 doi:10.1002/art.24264 19180514

[112] Clark A.R. and Dean J.L. (2012) The p38 MAPK pathway in rheumatoid arthritis: a sideways look. Open Rheumatol. J. 6, 209–219 doi:10.2174/1874312901206010209 23028406 PMC3460412

[113] Brook M., Sully G., Clark A.R. and Saklatvala J. (2000) Regulation of tumour necrosis factor α mRNA stability by the mitogen-activated protein kinase p38 signalling cascade. FEBS Lett. 483, 57–61 doi:10.1016/S0014-5793(00)02084-6 11033356

[114] Rahman M.M., Rumzhum N.N., Hansbro P.M., Morris J.C., Clark A.R., Verrills N.M. et al. (2016) Activating protein phosphatase 2A (PP2A) enhances tristetraprolin (TTP) anti-inflammatory function in A549 lung epithelial cells. Cell. Signal. 28, 325–334 doi:10.1016/j.cellsig.2016.01.009 26820662

[115] Rahman M.M., Rumzhum N.N., Morris J.C., Clark A.R., Verrills N.M. and Ammit A.J. (2015) Basal protein phosphatase 2A activity restrains cytokine expression: role for MAPKs and tristetraprolin. Sci. Rep. 5, 10063 doi:10.1038/srep10063 25985190 PMC4434956

[116] Frasca D., Romero M., Landin A.M., Diaz A., Riley R.L. and Blomberg B.B. (2010) Protein phosphatase 2A (PP2A) is increased in old murine B cells and mediates p38 MAPK/tristetraprolin dephosphorylation and E47 mRNA instability. Mech. Ageing Dev. 131, 306–314 doi:10.1016/j.mad.2010.02.002 20219523 PMC3223388

[117] Frasca D., Romero M., Diaz A., Alter-Wolf S., Ratliff M., Landin A.M. et al. (2012) A molecular mechanism for TNF-α-mediated downregulation of B cell responses. J. Immunol. 188, 279–286 doi:10.4049/jimmunol.1003964 22116831 PMC3700394

[118] Korhonen R. and Moilanen E. (2014) Mitogen-activated protein kinase phosphatase 1 as an inflammatory factor and drug target. Basic Clin. Pharmacol. Toxicol. 114, 24–36 doi:10.1111/bcpt.12141 24112275

[119] Abraham S.M., Lawrence T., Kleiman A., Warden P., Medghalchi M., Tuckermann J. et al. (2006) Antiinflammatory effects of dexamethasone are partly dependent on induction of dual specificity phosphatase 1. J. Exp. Med. 203, 1883–1889 doi:10.1084/jem.20060336 16880258 PMC2118371

[120] Bhattacharyya S., Brown D.E., Brewer J.A., Vogt S.K. and Muglia L.J. (2007) Macrophage glucocorticoid receptors regulate Toll-like receptor 4-mediated inflammatory responses by selective inhibition of p38 MAP kinase. Blood 109, 4313–4319 doi:10.1182/blood-2006-10-048215 17255352 PMC1885507

[121] Furst R., Schroeder T., Eilken H.M., Bubik M.F., Kiemer A.K., Zahler S. et al. (2007) MAPK phosphatase-1 represents a novel anti-inflammatory target of glucocorticoids in the human endothelium. FASEB J. 21, 74–80 doi:10.1096/fj.06-6752com 17099067

[122] Joanny E., Ding Q., Gong L., Kong P., Saklatvala J. and Clark A.R. (2012) Anti-inflammatory effects of selective glucocorticoid receptor modulators (SGRMs) are partially dependent on upregulation of dual specificity phosphatase 1 (DUSP1). Br. J. Pharmacol. 165, 1124–1136 doi:10.1111/j.1476-5381.2011.01574.x 21718312 PMC3346253

[123] Quante T., Ng Y.C., Ramsay E.E., Henness S., Allen J.C., Parmentier J. et al. (2008) Corticosteroids reduce IL-6 in ASM cells via up-regulation of MKP-1. Am. J. Respir. Cell Mol. Biol. 39, 208–217 doi:10.1165/rcmb.2007-0014OC 18314542

[124] Wang X., Nelin L.D., Kuhlman J.R., Meng X., Welty S.E. and Liu Y. (2008) The role of MAP kinase phosphatase-1 in the protective mechanism of dexamethasone against endotoxemia. Life Sci. 83, 671–680 doi:10.1016/j.lfs.2008.09.003 18845168 PMC2600599

[125] Smoak K. and Cidlowski J.A. (2006) Glucocorticoids regulate tristetraprolin synthesis and posttranscriptionally regulate tumor necrosis factor alpha inflammatory signaling. Mol. Cell. Biol. 26, 9126–9135 doi:10.1128/MCB.00679-06 16982682 PMC1636823

[126] Prabhala P., Bunge K., Ge Q. and Ammit A.J. (2016) Corticosteroid-induced MKP-1 represses pro-inflammatory cytokine secretion by enhancing activity of tristetraprolin (TTP) in ASM cells. J. Cell. Physiol. 231, 2153–2158 doi:10.1002/jcp.25327 26825339

[127] Blackshear P.J., Phillips R.S., Ghosh S., Ramos S.V., Richfield E.K. and Lai W.S. (2005) Zfp36l3, a rodent X chromosome gene encoding a placenta-specific member of the tristetraprolin family of CCCH tandem zinc finger proteins. Biol. Reprod. 73, 297–307 doi:10.1095/biolreprod.105.040527 15814898

[128] Clement S.L. and Lykke-Andersen J. (2008) A tethering approach to study proteins that activate mRNA turnover in human cells. Methods Mol. Biol. 419, 121–133 doi:10.1007/978-1-59745-033-1_8 18369979

[129] Bell S.E., Sanchez M.J., Spasic-Boskovic O., Santalucia T., Gambardella L., Burton G.J. et al. (2006) The RNA binding protein *Zfp36l1* is required for normal vascularisation and post-transcriptionally regulates VEGF expression. Dev. Dyn. 235, 3144–3155 doi:10.1002/dvdy.20949 17013884

[130] Stumpo D.J., Byrd N.A., Phillips R.S., Ghosh S., Maronpot R.R., Castranio T. et al. (2004) Chorioallantoic fusion defects and embryonic lethality resulting from disruption of Zfp36L1, a gene encoding a CCCH tandem zinc finger protein of the tristetraprolin family. Mol. Cell. Biol. 24, 6445–6455 doi:10.1128/MCB.24.14.6445-6455.2004 15226444 PMC434251

[131] Stumpo D.J., Broxmeyer H.E., Ward T., Cooper S., Hangoc G., Chung Y.J. et al. (2009) Targeted disruption of Zfp36l2, encoding a CCCH tandem zinc finger RNA-binding protein, results in defective hematopoiesis. Blood 114, 2401–2410 doi:10.1182/blood-2009-04-214619 19633199 PMC2746470

[132] Galloway A., Saveliev A., Łukasiak S., Hodson D.J., Bolland D., Balmanno K. et al. (2016) RNA-binding proteins ZFP36L1 and ZFP36L2 promote cell quiescence. Science 352, 453–459 doi:10.1126/science.aad5978 27102483

[133] Hodson D.J., Janas M.L., Galloway A., Bell S.E., Andrews S., Li C.M. et al. (2010) Deletion of the RNA-binding proteins ZFP36L1 and ZFP36L2 leads to perturbed thymic development and T lymphoblastic leukemia. Nat. Immunol. 11, 717–724 doi:10.1038/ni.1901 20622884 PMC2953641

[134] Hyatt L.D., Wasserman G.A., Rah Y.J., Matsuura K.Y., Coleman F.T., Hilliard K.L. et al. (2014) Myeloid ZFP36L1 does not regulate inflammation or host defense in mouse models of acute bacterial infection. PLoS ONE 9, e109072 doi:10.1371/journal.pone.0109072 25299049 PMC4192124

[135] Benjamin D., Schmidlin M., Min L., Gross B. and Moroni C. (2006) BRF1 protein turnover and mRNA decay activity are regulated by protein kinase B at the same phosphorylation sites. Mol. Cell. Biol. 26, 9497–9507 doi:10.1128/MCB.01099-06 17030608 PMC1698544

[136] Maitra S., Chou C.-F., Luber C.A., Lee K.-Y., Mann M. and Chen C.-Y. (2008) The AU-rich element mRNA decay-promoting activity of BRF1 is regulated by mitogen-activated protein kinase-activated protein kinase 2. RNA 14, 950–959 doi:10.1261/rna.983708 18326031 PMC2327367

[137] Schmidlin M., Lu M., Leuenberger S.A., Stoecklin G., Mallaun M., Gross B. et al. (2004) The ARE-dependent mRNA-destabilizing activity of BRF1 is regulated by protein kinase B. EMBO J. 23, 4760–4769 doi:10.1038/sj.emboj.7600477 15538381 PMC535089

[138] Graham J.R., Hendershott M.C., Terragni J. and Cooper G.M. (2010) mRNA degradation plays a significant role in the program of gene expression regulated by phosphatidylinositol 3-kinase signaling. Mol. Cell. Biol. 30, 5295–5305 doi:10.1128/MCB.00303-10 20855526 PMC2976374

[139] Blackshear P.J. and Perera L. (2014) Phylogenetic distribution and evolution of the linked RNA-binding and NOT1-binding domains in the tristetraprolin family of tandem CCCH zinc finger proteins. J. Interferon Cytokine Res. 34, 297–306 doi:10.1089/jir.2013.0150 24697206 PMC3976581

[140] Adachi S., Homoto M., Tanaka R., Hioki Y., Murakami H., Suga H. et al. (2014) ZFP36L1 and ZFP36L2 control LDLR mRNA stability via the ERK-RSK pathway. Nucleic Acids Res. 42, 10037–10049 doi:10.1093/nar/gku652 25106868 PMC4150769

